# Associations of maternal smoking during pregnancy with academic performance in adolescent offspring: findings from a registry data-based cohort study

**DOI:** 10.1192/j.eurpsy.2023.211

**Published:** 2023-07-19

**Authors:** G. Ayano, B. Dachew, K. Betts, R. Alati

**Affiliations:** School of Population Health, Curtin University, Perth, Australia

## Abstract

**Introduction:**

Emerging epidemiological data have indicated associations between maternal smoking during pregnancy and a range of negative outcomes in children. Nevertheless, there is scant evidence reporting adverse effects on lower academic performance during adolescence.

**Objectives:**

To examine the association between maternal smoking during pregnancy and the risk of lower academic performance in adolescent children.

**Methods:**

Data were obtained from the New South Wales (NSW) Perinatal Data Collection, which included all live births in the Australian state of NSW from January 2003 to December 2005. This was linked with NSW admitted data collection and National Assessment Program for Literacy and Numeracy (NAPLAN). A total of 168, 528 mother-offspring pairs were involved in the final analysis. Maternal smoking during pregnancy was assessed using self-reports of smoking during pregnancy. NAPLAN was used to assess the educational performance of the offspring. A logistic regression model was used to explore the association.

**Results:**

The findings show that exposure to cigarette smoke in utero was associated with an increased risk of poor academic performance in adolescent offspring in all domains, including numeracy [OR, 2.43 (95%CI 2.30-2.58)], reading [OR, 2.49 (95%CI 2.37-2.62)], writing [OR, 2.97 (95%CI 2.84-3.11)] and spelling [OR, 3.12 (95%CI 2.98-3.26)]. In our sensitivity analysis by gender, maternal smoking during pregnancy demonstrated stronger effects on the academic achievements of females in all domains.

**Conclusions:**

The results show that exposure to cigarette smoke in utero was associated with an increased risk of lower educational achievements in adolescent children with greater effects in female than male children in all domains. The findings suggest the potential for targeted screening and early intervention of academic performance in exposed offspring.

**Disclosure of Interest:**

None Declared

